# Contrasting approaches to ‘doing’ family meals: a qualitative study of how parents frame children’s food preferences

**DOI:** 10.1080/09581596.2015.1089353

**Published:** 2015-10-01

**Authors:** Claire Thompson, Steven Cummins, Tim Brown, Rosemary Kyle

**Affiliations:** ^a^ Faculty of Public Health & Policy, Department of Social & Environmental Health Research, London School of Hygiene & Tropical Medicine, London, UK; ^b^ School of Geography, Queen Mary University of London, London, UK; ^c^ Sandwell PCT, West Bromwich, UK

**Keywords:** Family meals, parents, food provisioning, eating routines, qualitative study

## Abstract

Family meals, as acts of domestic food provisioning, are shaped by the competing influences of household resources, food preferences and broader cultural norms around dietary practices. The place of children’s food tastes in family meal practices is particularly complex. Food tastes stand in a reciprocal relationship with family food practices: being both an influence on and a product of them. This paper explores how parents think about and respond to their children’s food preferences in relation to family meal practices. A qualitative study was conducted with residents of Sandwell, UK. The results presented here are based on the responses of nine key participants and their families. Photo elicitation methods generated participant food photo diaries that were used to inform subsequent interviews. A thematic analysis revealed two contrasting ways of incorporating children’s tastes into family meal routines: (1) ‘what we fancy’ and (2) ‘regulated’. The former entails repeatedly consulting and negotiating with children over what to cook for each meal. It is supported by the practical strategies of multiple and individually modified meals. The latter relies upon parents developing a repertoire of meals that ‘work’ for the family. This repertoire is performed as a series of ‘set meals’ in which any requests for variation are strongly resisted. Our findings add to the small body of literature on household food provisioning and suggest that achieving the idealised ritual of the family meal is underpinned by a range of values and strategies, some of which may run counter to health messages about nutrition.

## Introduction

A meal is a physical event that must be prepared for and one that is tightly bound by social rules and roles (Bell & Valentine, 1997). A ‘proper’ family meal, in this context, can be understood as one for which *all* family members are present, sit down and take their time over a shared food, prepared in the home and eaten together (Charles & Kerr, 1988). Family meals, as acts of domestic food provisioning, are shaped by the competing influences of household resources, food preferences, and broader socio-cultural norms around dietary practices and rituals (Schubert, 2008). Therefore, how mealtimes are performed is the result of a complex interplay of context, constraints, values, compromises, tastes and identities. Understanding variations in the internalised logic, which informs family meal practices is a useful way of exploring this interplay (Delormier, Frohlich, & Potvin, 2009; Schubert, 2008). This paper examines one particular aspect of these intersecting influences: specifically, how parents frame and respond to the food preferences and tastes of their children in the context of family mealtimes. Children’s food tastes can exert considerable influence on family food practices and yet, at the same time, these tastes are also situated in and shaped by the specific socio-cultural family context in which they are performed. Food tastes are embodied and emplaced practices (Beagan et al., 2015).

Negotiation between parents and children over the content of meals can be an ongoing power struggle around the privileging of tastes. Women report prioritising their partners and children’s tastes over their own because it is seen as a way of both avoiding wasting food and expressing love. Routinely incorporating children’s preferences can become a strategy to ensure the participation of children in family meal rituals (Charles & Kerr, 1988; Meah, 2013). Mealtimes are then the site of potential conflict, where the tensions between socialisation and individual tastes are played out and negotiated by parents (Charles, 1995). They must navigate this intersection between aspiration and actuality; between the cultural ideal of the family meal and its practical performance with children (Charles & Kerr, 1988; Jackson, Olive, & Smith, 2009).

The extent to which parents routinely engage in negotiation over meal content depends upon how they frame their children’s food preferences in relation to the domestic task of feeding the family. Whilst children can be treated as simply passive receivers of food, they can also be facilitated as active participants in the process of determining what is eaten by the whole family (Carrigan, Szmigin, & Leek, 2006; Wills, Backett-Milburn, Gregory, & Lawton, 2008). Literature on feeding the family has been critiqued for failing to address complex intrafamilial processes such as these, which in practice directly affect consumption (Campbell, 1995).

Whilst the influence of children’s food preferences on family meals is extensively addressed in behavioural nutrition research on parental feeding styles (Patrick, Nicklas, Hughes, & Morales, 2005), there is an absence of research that examines them in the context of food provisioning, the values that underpin them and the process of arriving at a set of food practices (Schubert, 2008). Equally, there is little empirical research that adopts a micro-level perspective on patterns of thinking and behaviour around food work that contribute to the maintenance of the family and the household (Moisio, Arnould, & Price, 2004). This paper addresses these gaps using photo elicitation interview data from a qualitative study to explore how parents routinely think about and respond to their children’s food preferences in relation to family meal practices.

## Methods

### Recruitment and sampling

A qualitative study of food behaviours was carried out over a six-month period in 2010 in Sandwell, a metropolitan borough in the West Midlands. Sandwell has a population of approximately 292,800 (Sandwell PCT, 2010). It is the 12th most deprived local authority in England and has comparatively high levels of unemployment, benefit claims, teenage pregnancy, smoking, obesity and limiting long-term illnesses (Black Country Consortium, 2011; NHS, 2011; Sandwell PCT, 2010). Participants were recruited from community settings including community centres, primary schools and libraries through introductions made by local gatekeepers. Where possible, participants were briefed and interviewed in these community settings or in their homes. As part of informed consent it was explained that participants had the right to withdraw from the study at any time and that all data would be de-identified. Permission to record interviews was also sought, and digital cameras (with instructions) were issued to each participant. At the end of the study, participants were allowed to keep their camera. This proved to be an effective way of engaging and retaining people.

The findings presented here are based on the responses of a subset of participants from the original study. This subset comprised nine key participants who were selected specifically because they were parents of young and/or school age children. These participants were invited to include their partners and children in the interview and data collection activities if they felt comfortable doing so. In total, seven children and three partners attended interviews at various points (which, along with the nine key participants, made a total of 19 overall). The characteristics of the nine key participants and their families are described in Table 1.

**Table 1.** Key participant characteristics.

Pseudonym	Social characteristics	Reported household composition and food practices
Melissa	Female, African-Caribbean, 47 years, Employed	Lived with her secondary school-age daughter. Her adult son visited several times a week and cooked most of the meals. Erratic eating patterns due to her shift work. Very accommodating of individualised practices
Sue^a^	Female, White British, 49 years, Not in paid employment	Lived with her twin secondary school age daughters^c^. Her partner stayed with them regularly and his son (12 years old) visited at weekends. Often cooked multiple meals per sitting with the exception of a ‘proper Sunday dinner’
Collette^a^	Female, White British, 44 years, Not in paid employed	Lived with her five children^c^, three of whom were at secondary school, one in college and one at work. Reported a great deal of flexibility and reactivity. Food shopping was particularly complex and sometimes fraught
Jayanti^a^	Female, British Indian, 45 years, Employed	Lived with her husband^b^, who worked full-time, and two of their children (both at secondary school). Her eldest daughter was at university and sometimes came home at weekends. Observed some Hindu dietary practices depending on context. Husband reported ‘breaking’ with them, especially when at work
Hasan^a^	Male, Bangladeshi, 34 years, Employed	Lived with his wife (Mahida), who stayed at home with their five children, all under the age of seven. His mother, a vegetarian, also lived with them. Regulated approach with provisioning tasks divided clearly. Observed Halal at home
Lisa^a^	Female, White British, 38 years, Employed	Lived with her husband (Derek^a^^,^^b^), who worked full-time, and their three children. One child was nursery-aged^c^ and two were at primary school. Highly organised. Lisa often worked from home
Catherine	Female, White British, 37 years, Employed	Lived with her husband, who worked full-time, and their nursery age daughter^c^. Mostly flexible but with some regulated elements. Self-identified as a ‘healthy eater’ and vegetarian
Poppy	Female, White British, 43 years, Not in paid employment	Lived with her husband^b^, who worked full-time, and their nursery-age daughter^c^. She cooked all meals and described her husband as ‘easy going’. Regulated with some elements of individual catering. Self-identified as an ‘ethical consumer’ and vegetarian
Caroline^a^	Female, White British, 52 years, Employed	Lived with her husband, who worked full-time, and their two sons, one of whom had just started work; the other was at secondary school. Very individualised. Mother and both sons self-identified as ‘picky eaters’

^a^ Quoted in the paper.

^b^ Partner who was also present at interview (three in total).

^c^ Child who was also present at interview (seven in total).

The first author conducted all the interviews and met with every key participant at least four times, spending a total of around three or four hours with each of them.

### Photo elicitation data collection

Empirically exploring everyday practices, like family meals, requires the use of methods that can explore tacit aspects of household food provisioning and contextual factors such as identity and socio-cultural background (Wills, 2012). The flexible, taken-for-granted nature of everyday realities means they are often carried out without reflection or fuss and are therefore less accessible to researchers using traditional interview methods (O’Connell, 2013).

Visual methods are well suited to investigating aspects of everyday life that are difficult to otherwise put into words, and can represent knowledge more forcefully and richly (Power, 2003). Participant-produced photographs, in particular, provide a far deeper sense of experiences and world views than could be expressed simply in words; they can explore habitus by uncovering the contingencies and improvisations that characterise everyday life (O’Connell, 2013; Sweetman, 2009).

The present study used photo-elicitation methods to collect data and structure interviews. Photo-elicitation is based on the premise of inserting photographs into research interviews (Oliffe & Bottorff, 2007). The addition of participant-produced photographs allows the researcher to access a more detailed account of participant practices and, in this case, explore the nuances of family food decision-making. Over a four-day period participants were asked to photograph everything they ate and drank, where this took place and with whom. Participants were issued with digital cameras and supporting materials in order to undertake this activity. The aim was to compile a detailed ‘what, where and who with’ representation of eating habits to elicit representations of their embedded family food practices (Sharma & Chapman, 2011). The photographs served as visual diaries that were then used to prompt and inform subsequent interviews (Dennis, Gaulocher, Carpiano, & Brown, 2009). Keeping visual records served to highlight contradictions and context-specific behaviours. The interview schedule was developed to elicit a narrative account of the four-day period by progressing through the photographs in sequence, encouraging participants to tell the ‘story’ of their food practices and generating descriptions of ‘typical’ routines. For example, the prompt ‘Is this what you normally eat on this day/at this time/when at work etc.?’ was used repeatedly to establish routine behaviours.

Interpretation and levels of engagement for the photo-elicitation activity differed significantly. The number of photographs per photo-diary ranged from 15 to 75. Some participants took the time to clearly record the exact contents of the photographs and the times they were taken, whereas others just took photographs of the meals they ate and did not include contextual information. Decisions about what to photograph and what counts as ‘relevant’ are interpretive decisions made by participants (Harper, 2002).

### Data analysis

All interviews were audio recorded and transcribed verbatim. The NVivo9 software package was used to support a thematic analysis, a type of analysis that seeks to identify patterns of experience, talk and behaviour (Aronson, 1994). Open coding was used to identify and categorise eating episodes, social context, accounts of food choices and descriptions of repetitive food practices. Selective coding was used to identify the values and motivations that linked codes and informed ‘bundles’ of practices. Transcripts were then re-examined for contradictions, dilemmas and omissions. Finally, a coding frame was developed and refined to capture the main concepts. The coding frame helped to establish themes describing sets of consistent practices around mealtimes that could be described as ‘routine’. Two distinct ways of framing and responding to children’s food preferences emerged from the analysis and are described below.

## Results

In this section, we explore how the tastes and preferences of children were framed and managed by parents and how this contributes to the negotiated accomplishment of family meals. Our analysis suggests that there are two contrasting ways of incorporating children’s tastes that parents adopted when providing family meals: ‘what we fancy’ and ‘regulated’. The characterisation of these approaches is related to practices and performances of household food provisioning tasks, rather than the qualities and characteristics of the individuals who performed them (Halkier & Jensen, 2011). Thus, the two approaches are not intended to represent binary and static caricatures of the parents that deployed them. Rather, they are ways of thinking and strategising that parents draw upon in order to ‘do’ family meals in different ways. Both approaches are described in relation to two main criteria: the reported nature and extent of parents’ negotiation over what foods will be consumed; and the specific strategies parents employed to ensure successful meals.

### ‘What we fancy’: individually modified and multiple meals

**Figure 1.**
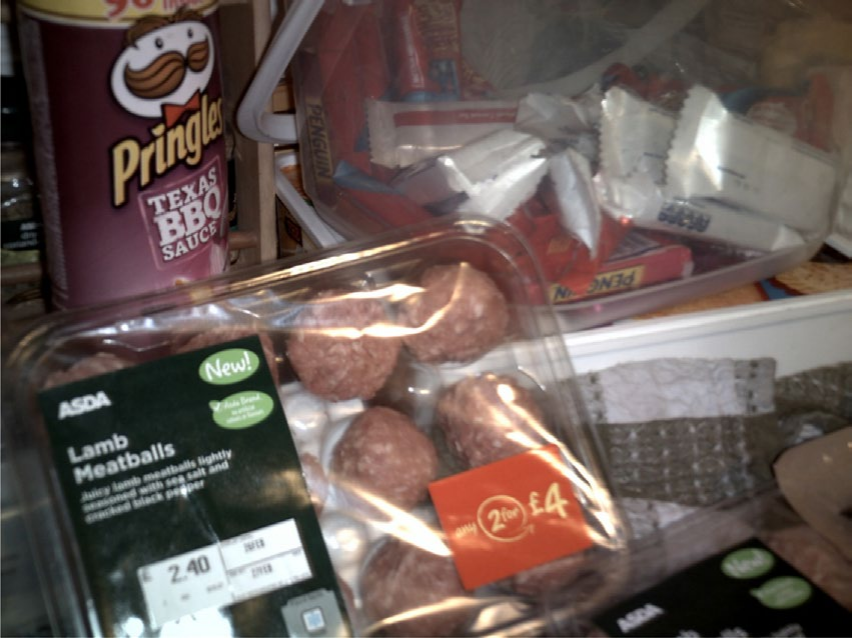
Collette’s photograph of ‘meatballs night’ ‘meatballs’.

**Figure 2.**
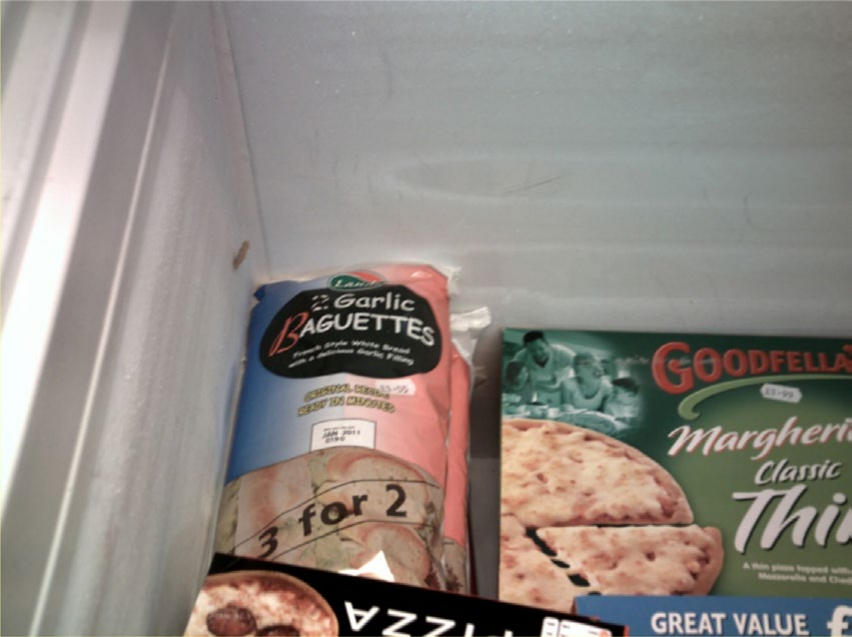
Collette’s photograph of ‘meatballs night’ ‘garlic bread’.

**Figure 3.**
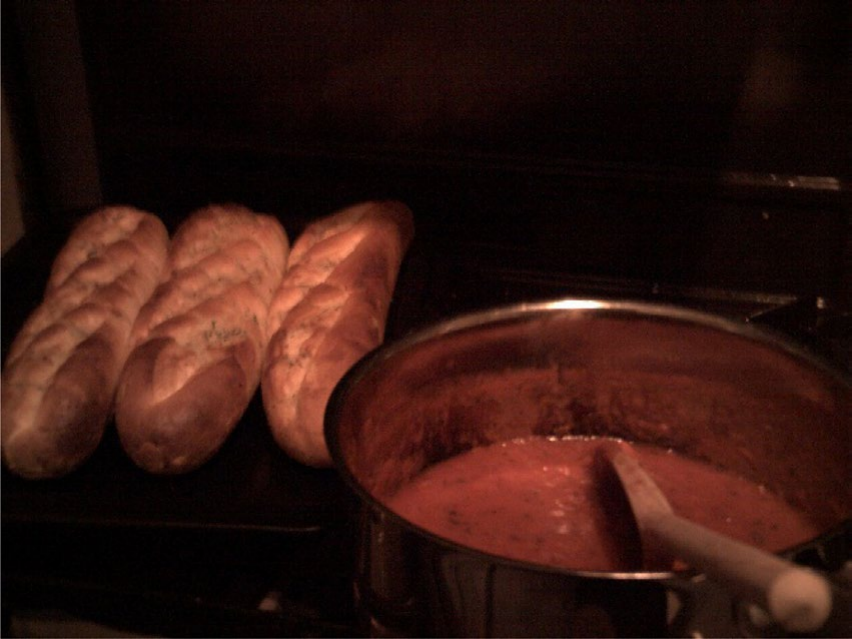
Collette’s photograph of ‘meatballs night’ ‘sauce and garlic bread’.

**Figure 4.**
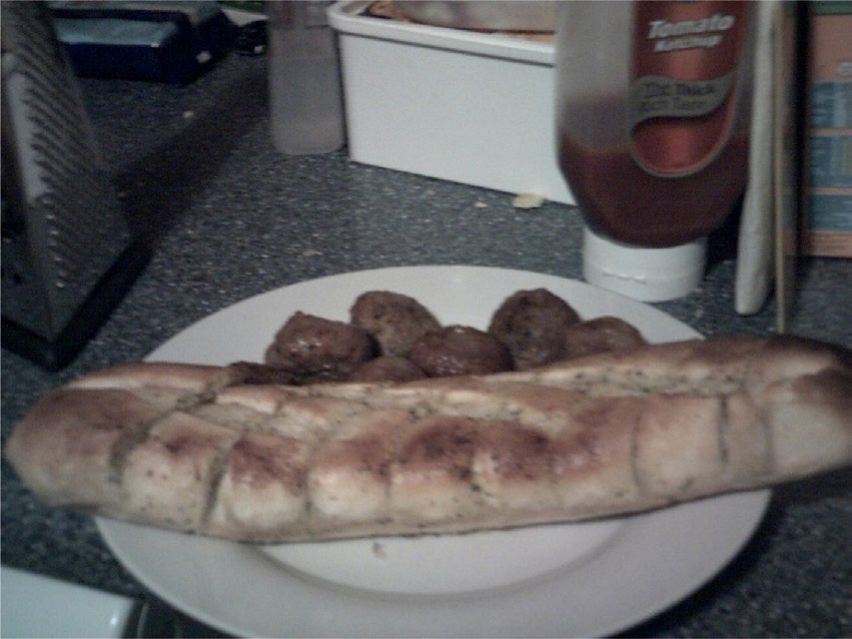
Collette’s photograph of ‘meatballs night’ ‘meatballs and garlic bread’.

This approach is so named because the participants who employed it frequently explained meal practices in terms of what family members ‘fancied’ eating at each sitting. This set of practices revolved around continually negotiating the content and form of family meals with children. Typically, participants reported consulting their children before preparing most meals and attempting to reach an agreement that was acceptable to all members of the family. In this sense, the achievement of family meals was constructed as a collaborative process, with children positioned as active participants in the formulation and performance of mealtimes. This feature is aptly illustrated by Collette, a mother of five, who recounts the interactions that led up to the production and consumption of the family meal depicted in her photographs (see Figures 1–4: ‘meatballs night’).

Collette: This was meatballs night (pointing at photograph). And it ended up with no pasta, just the meatballs, a sauce which was a jar plus shopped tomatoes [pause] so I did add some fresh to it, and garlic bread, which I suppose was OK but to me isn’t very filling. I mean I didn’t have the meatballs, I just had the sauce but I didn’t do myself some pasta to go with it. But it ruined it for me cos I was looking forward to something … I even grated the cheese ready to put on top of the meatballs but it just didn’t happen.

Interviewer: Why didn’t it happen?

Collette: Well, they had no enthusiasm for it. ‘I’ll do some pasta.’ ‘No.’ ‘I’ll do some meatballs.’ ‘No’. ‘Well eat them now I’ve cooked them.’ ‘OK.’

Interviewer: So who ate that then?

Collette: Martin (son) didn’t. Well actually he ate garlic bread and sauce and he had grated cheese on his, he did have that. But Vince (son), Dawn (daughter) and Sara (daughter) did have the meatballs and that so [pause] I had more or less the same as Martin. Meatballs had to be eaten though, cos as I said they’d been in the freezer for too long and they needed to be cooked cos I needed room for the next time I did the shopping (laughs). But er … it was an idea I’d had and I kept bringing it up every night. ‘Shall we do the meatballs tonight?’ ‘No.’ ‘Shall we do the meatballs tonight?’ ‘No.’ [pause] So [pause] but that failed … and I do, I ask them for ideas.

Collette recounts a complex set of negotiations with her children, directly reporting the verbal exchanges between them and her efforts to cater to everyone’s individual tastes and muster ‘enthusiasm’ for the meal. She recalled being very disappointed that her children’s tastes prevented her from cooking the meal in a much more straightforward manner. Collette reported regularly modifying family meals to suit her children’s preferences at the expense of her own. As can be seen above, she made a number of additions and compromises to ensure that the meal suited everyone’s tastes, such as cooking garlic bread instead of pasta. As a result of consulting with her children as to their individual preferences she varied the meal according to their tastes. She diffused tension over the choice and content of the meal by deferring to the tastes of her children (Charles & Kerr, 1988; DeVault, 1991; Meah, 2013). Interestingly, the photographs she presented depict the component parts of the meal as she selected and prepared them, and only one photograph of the meal ready to serve (Figure 4). This reflects the negotiated and malleable nature of meal formats in these practices. As Collette states, she had repeatedly suggested the meatballs-meal to her children over the last few weeks and regularly asked them for meal ideas.

Facilitating children as active agents in meal practices can mean ongoing disagreements and resistance (Carrigan et al., 2006; Charles, 1995). Family meals can be fraught with conflict, bickering and power struggles with children who may be unwilling to participate and rapidly asserting their own tastes and practices (O’Connell, 2014; Wills, Appleton, Magnusson, & Brooks, 2008). Sue, a mother of twin girls, summed up the rationale for this strategy neatly when she explained ‘I’d rather them eat something they want cos if I do them something they don’t want, they ain’t going to eat it are they?’ Individually modified meals are a strategic compromise, a trade-off in food provisioning.

The other main strategy employed in this approach was that of providing multiple meals for each sitting. If modifying aspects of the family meal, as described above, failed to meet everyone’s preferences adequately, parents also adopted the strategy of preparing separate meals for each of their children. This tended to necessitate heavy reliance on frozen and/or pre-prepared foods. By doing this, parents could both address diverse tastes and, at the same time, ensure that all the family sat down together to enjoy a shared mealtime (if not an actual shared meal). This strategy was a practical means of offsetting their children’s individual preferences against the collective nature of the meal ritual. Caroline, a mother of two teenage sons, reported routinely providing multiple meals:

I even cook three or four different meals some nights … because we’re all so fussy I think ‘right we haven’t had this for a while, we’ll have this.’ Well, you know cos it can get very samey can’t it. I mean they don’t say anything but sometimes they can be a bit ‘oh’. I’ll go ‘right, I’m doing so and so.’ ‘Are we having that again?’ ‘OK’ then I’ll do something different. But you run out of ideas, especially with … when you’ve got to make not just one meal but you’re doing two or three.

As Caroline explains, multiple meals are a practical response to the ‘fussiness’ of her family.

The trend towards parents preparing multiple meals ‘on demand’ reflects the increasingly flexible nature of contemporary family life and the individualistic tendencies that entails (Wills, Appleton et al. 2008). Progressively more fragmented and individualised social lives, coupled with changes in the profile of the labour force, has resulted in a greater diversity in family relationships and routines. Tailoring foods to accommodate individual preferences is now a common practice at family mealtimes (DeVault, 1991). It is a way of showing love and reproducing relationships within the family (Moisio et al., 2004).

Both Caroline and Collette’s accounts feature substantial amounts of reported speech. They describe the back and forth between themselves and their children about the content of their meals. Their suggestions, reactions and modifications are all described. This was a common narrative feature of the ‘what we fancy’ approach, and one that sometimes made accounts difficult to follow. Parents did not just speak for themselves; they also reported the utterances and influences of their partners and children. The consultative nature of this approach, in which children’s individual tastes are framed as an integral part of deciding what to eat at mealtimes, is reflected in the dialogic quality of the descriptions. This framing is reproduced by the practical strategies of individually modified meals and multiple meals.

### ‘Regulated’: set meal repertoires and resisting variations

This approach entailed an alternative framing of children’s preferences and assigned them a less prominent role in ‘doing’ family meals. The achievement of shared family meals, in which the family all ate the same foods together, was described by parents as their overall aim. In this case, rather than negotiate with their children at each mealtime the parents deploying this approach developed a repertoire of set meals that could be shared by the whole family. To a degree, these meals incorporated the individual likes and dislikes of family members. However, in contrast to the ‘what we fancy’ approach, children’s tastes were by no means the primary consideration. There was very little negotiation *at* mealtimes. Instead, the collaborative aspects of this approach were located firmly in parents’ development of a range of acceptable meals that they thought ‘worked’ for the family. When preparing and performing these meals for the family, parents reported tolerating very little resistance from children and actively resisted requests for individual modifications.

Eating the same foods was crucially important to performing ‘proper’ meals. For example, Lisa and Derek, a couple with three children, described the nightly family meal as a fundamental part of bringing their children up ‘properly’. They performed this in a very structured way. Lisa did all the food shopping and Derek did all the cooking. Over the years, the couple had compiled their own home-made recipe book that they continually added to with ‘things that work’, a repertoire of family meals that they felt to be sufficiently practical, enjoyable and healthful and meant that the whole family could eat the same food. In the mornings, Lisa would pick out a recipe and make sure the relevant ingredients were laid out in the kitchen by early evening. When Derek returned home from work, he would look to see which recipe Lisa had selected and prepare the evening meal accordingly. Once they had decided on a meal and prepared it they did not welcome disruptions or requests to modify the meal for individuals. In the following extract, the couple explain how they dealt with the disruption of the Derek’s brother coming over to dinner, as he had very particular tastes:

Lisa: But I hate doing that (cooking more than one meal). I [pause] I don’t allow my children to do that. If we’re having beef that’s what we’re having. So if his (Derek’s) brother comes over and we do separately for him then it’s a bad message for my kids …

Interviewer: Do you have him over for dinner a lot?

Lisa: No (laughs) … Or if we do you know we won’t do beef we’ll do chicken or something else.

Derek: We’ll do chicken or something so it doesn’t look like we’ve given in.

They disliked the ‘message’ that cooking multiple meals or catering to individual preferences sent to their children. It might look as if they had ‘given in’ by allowing individual demands to dictate what was cooked. The inclusion of Derek’s brother in the family meal ritual made it more difficult to adhere to the ideal of achieving a shared meal (as opposed to merely a shared *mealtime*) and avoid openly negotiating meal content in front of their children. Family food consumption through structured ‘proper’ meals, as Lisa and Derek describe, socialises children into what are considered appropriate food practices, social behaviours and identities (Charles & Kerr, 1988). They valued their established meal rituals and repertoires. They employed the practical strategies of set meals and resisting individual practices in order to maintain them. In this approach, requests for variations at mealtimes were judged very negatively and resisted, even to the point of avoiding certain guests. Mealtime rituals, in this context, functioned as a kind of regulatory mechanism – as a way of maintaining a coherent family ideology (Charles & Kerr, 1988; Grieshaber, 1997).

Hasan, a father of five young children, and his wife, Madiha, also used the strategy of developing set meal repertoires. As in the previous example, they also routinely divided food provisioning tasks between them to achieve this. Hasan did all the food shopping, bulk buying once a week, whilst Madiha prepared all the meals. As he explains, the family employed a ‘timetabled’ method of food provisioning:

[The] days are all set out when she (Madiha) knows what to do. It’s all timetabled the food because obviously, having a big family [pause]. When you’ve got kids, mum, mother-in-law at home, you have to make sure most of the food is prepared…. So, yeah, she’s really organised. Plus we get, like, relatives popping in as well.

This careful pre-planning contrasts with the reactivity of catering to what individuals ‘fancy’ eating in that it does not lend itself to consultation and negotiation at mealtimes. Children’s input and influence is less direct and immediate compared with the previous approach. Parents incorporate their children’s preferences and feedback into subsequent versions of the meals and in the formats that become part of their household repertoire of ‘things that work’. Unlike the ‘what we fancy’ approach, they do not invite their input when performing mealtimes and they typically refuse requests for individual modifications to meals.

The rationale for these strategies is both practical and ideological. On a practical level parents explain that adhering to a set meal format from their family repertoire avoids waste, saves time and money and means that they do not have to undertake what they consider to be ‘too much’ cooking. Jayanti, a mother of three who kept to a rigid set of family meal practices, was very clear about this when explaining how she delivered family meals:

Interviewer: And do you always eat the same things at meal times?

Jayanti: Yeah. Yeah. I make them … I’m not cooking different things.

She went on to elaborate:

When our oldest son [pause] he’s a bit picky. He’s the one that’s a bit, I told you about [pause]. He likes … even though he’s like a match stick he prefers meat and I say ‘no, you’re not having it all the time’.

**Figure 5.**
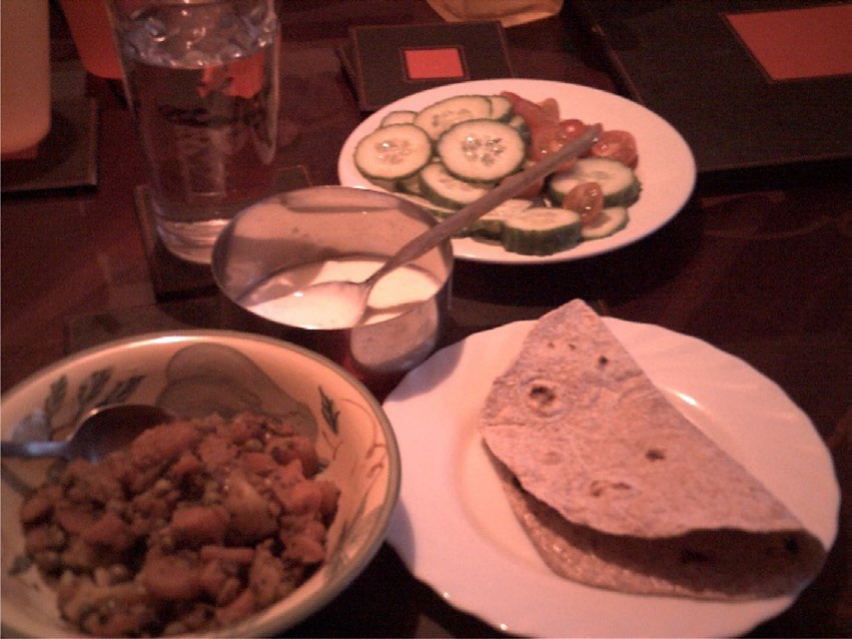
Jayanti’s photograph of a vegetarian family meal: ‘lentil curry and bits’.

**Figure 6.**
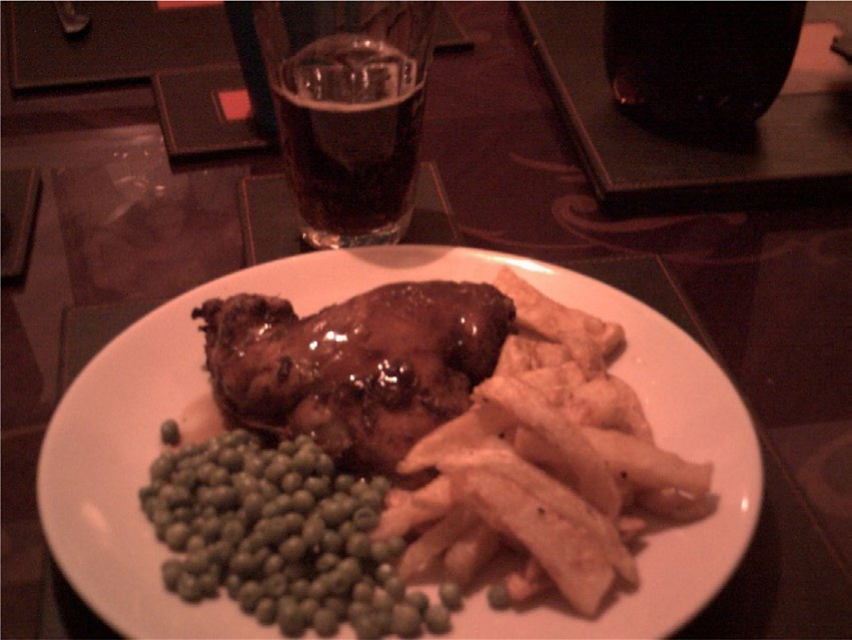
Jayanti’s photograph of a meat-based family meal: ‘chicken and chips’.

Jayanti cooked meals with meat on four days of the week and vegetarian meals on the remaining three days (see Figures 5 and 6). As she stated, despite her oldest son’s preference for meat, she refused to deviate from this practice at his request. He was given meat, but only on days when the whole family ate meat together as part of a shared family meal. These strategies served to instil what parents considered to be ‘appropriate’ eating habits by requiring that children eat the foods that parents chose. As Lisa put it, sometimes it was necessary to exercise ‘tough love’ in this respect. Achieving a shared meal was valued above catering to individual tastes and requests at mealtimes. As can be seen in her photographs, the images Jayanti captured simply presented the meals dished up and ready to be consumed – as finished products. Unlike Collette’s photo-diary, Jayanti’s did not include photographs of ingredients or any preparation of food stuffs. Instead, it focused exclusively on the finished meals, reflecting an emphasis on achieving shared meals rather than responding to requests.

## Conclusion

In this paper, we have contributed to the body of research on household food provisioning by examining the specific ways in which parents think about and respond to their children’s food preferences. The two approaches represented differing ways of routinely managing children’s tastes and therefore result in differing performances of family meal rituals. ‘What we fancy’ represented a more reactive approach, for which parents tended to consult with their children at mealtimes. The ‘regulated’ approach did attach value to children’s tastes, but to a lesser extent and in a less immediate fashion. Parents incorporated their family’s preferences in the development of their meal repertoires, but did not welcome input or negotiation *at* mealtimes.

The focal point of these findings is the perceptions and strategies of parents. As a result, we have not addressed the views and opinions of children in their own words or actively examined how negotiations with parents unfold in real time. Such an undertaking would be a valuable addition to the work. These approaches certainly cannot be regarded as a comprehensive or exhaustive examination of the myriad variations on family meal rituals. They do, however, provide empirical data on social reproduction at the micro-level; they explore patterns of thinking and behaviour that ensure the integration of family members (particularly children) into the wider macro-social order (Moisio et al., 2004).

Investigating these practices is critical to understand dominant trends in the food system, such as individualised consumption or a tendency to prepare meals ‘on demand’. The emphasis of public health policy on discrete behaviours rather than embedded practices underplays the material and social limitations on ‘healthy eating’ (Attree, 2006). Public health nutrition policy and intervention needs to recognise the way eating habits are defined by and reproduce social and cultural capital (Schubert, 2008). Halkier and Jensen (2011) argue for a focus on sets of practices, rather than on individual actors. One way of addressing this is to extend the relatively small body of research of food provisioning by further investigating routine food practices. Whilst routine practices are a useful way to characterise whole diets, they remain relatively unused in public health research and health promotion (Jastran, Bisogni, Sobal, Blake, & Devine, 2009). Further research into the nutritional differences associated with varying mealtime routines is needed in order to identify the implications for dietary health.
